# Comparisons of Auditory Performance and Speech Intelligibility after Cochlear Implant Reimplantation in Mandarin-Speaking Users

**DOI:** 10.1155/2016/8962180

**Published:** 2016-06-16

**Authors:** Chung-Feng Hwang, Hui-Chen Ko, Yung-Ting Tsou, Kai-Chieh Chan, Hsuan-Yeh Fang, Che-Ming Wu

**Affiliations:** ^1^Department of Otolaryngology, Kaohsiung Chang Gung Memorial Hospital and Chang Gung University College of Medicine, Kaohsiung 83301, Taiwan; ^2^Department of Otolaryngology, Chang Gung Memorial Hospital and Xiamen Medical Center, Fujian 361000, China; ^3^Department of Otolaryngology, Linkou Chang Gung Memorial Hospital, College of Medicine, Chang Gung University, Taoyuan 33305, Taiwan

## Abstract

*Objectives*. We evaluated the causes, hearing, and speech performance before and after cochlear implant reimplantation in Mandarin-speaking users.* Methods*. In total, 589 patients who underwent cochlear implantation in our medical center between 1999 and 2014 were reviewed retrospectively. Data related to demographics, etiologies, implant-related information, complications, and hearing and speech performance were collected.* Results*. In total, 22 (3.74%) cases were found to have major complications. Infection (*n* = 12) and hard failure of the device (*n* = 8) were the most common major complications. Among them, 13 were reimplanted in our hospital. The mean scores of the Categorical Auditory Performance (CAP) and the Speech Intelligibility Rating (SIR) obtained before and after reimplantation were 5.5 versus 5.8 and 3.7 versus 4.3, respectively. The SIR score after reimplantation was significantly better than preoperation.* Conclusions*. Cochlear implantation is a safe procedure with low rates of postsurgical revisions and device failures. The Mandarin-speaking patients in this study who received reimplantation had restored auditory performance and speech intelligibility after surgery. Device soft failure was rare in our series, calling attention to Mandarin-speaking CI users requiring revision of their implants due to undesirable symptoms or decreasing performance of uncertain cause.

## 1. Introduction

Cochlear implantation (CI) is widely considered a standard and safe treatment for patients with severe-to-profound sensorineural hearing loss. However, complications still occur, which can sometimes lead to revision surgeries or even reimplantation. The CI complication rate is wide-ranging among institutions, from 2.3% to 8.0% for major complications and from 3.8 to 16.0% for minor complications [1]. In a recent review on CI revision surgery [2], the overall revision rate was 1.2–15.1% and the overall device failure rate was 0.5–14.7%. There are clearly large differences between medical institutions [2]. It is thus a vital issue that every cochlear implant center analyzes their own data related to complications and revisions to allow reexamination of clinical outcomes and to provide proper consultations to patients.

There are four types of revision surgery: reimplantation, minor revision surgery, explantation without reimplantation, and electrode array reinsertion [2]. The main reasons for reimplantation are device failure, device infection or extrusion, wound or flap complications, and upgrades of cochlear implant technology [3, 4]. Further classification into hard or soft device failure is defined by the guidelines of the 2005 Cochlear Implant Soft Failures Consensus Development Conference Statement [5]. According to those guidelines, “hard” failure refers to measurable hardware abnormalities and “soft” failure refers to declining performance, aversive auditory and nonauditory symptoms, or intermittent function but with maintained communication between external and internal components. Recently, reported rates of hard and soft failures have ranged from 0.6% to 5% and 0.6% to 7%, respectively [6–13].

In addition to the causes of device failure, outcomes after reimplantation surgeries also require close attention and regular follow-up evaluations. Many cochlear implant centers use the Categorical Auditory Performance (CAP) and the Speech Intelligibility Rating (SIR) scales to evaluate auditory and speech development in children with CI [14, 15]. However, auditory performance and speech intelligibility subsequent to reimplantation have not been discussed in Mandarin-speaking users [8, 12, 16, 17].

In this report, we review our experience with CI surgeries in Mandarin-speaking users over a 16-year period, emphasizing causes, auditory performance, speech intelligibility, and more difficult specific tone and speech perception tests after reimplantation.

## 2. Materials and Methods

### 2.1. Study Population

A retrospective review was carried out on 589 patients who underwent consecutive cochlear implant surgeries performed by two surgeons during the period from 1999 to 2014. One surgeon performed the surgeries in the Linkou branch (*n* = 401) of Chang-Gung Memorial Hospital, a tertiary medical center, and the other in the Kaohsiung (*n* = 140) and Xiamen branches (*n* = 48; Table 1). Collected data included the etiologies, age at implantation, type of device originally implanted, and types of complications that occurred. For those who underwent revision surgeries, we also reviewed their age at revision surgery, the interval between the implantation surgery and the revision surgery, the management (reimplantation/explantation), the cause of the revision surgery, the device newly implanted, and hearing and speech perception outcomes after reimplantation. This study protocol was approved by the institutional review board of Chang-Gung Memorial Hospital. All patient records/information were anonymized and deidentified prior to analysis.

### 2.2. Cochlear Implantation

*In this series*, CI was performed using a standard technique of cortical mastoidectomy and subsequent posterior tympanotomy via an inverted J incision (*n* = 255) or a minimally invasive incision (*n* = 334; CMW and CFH adopted after 2009 and 2005, resp.). All of the patients were fitted with an implant manufactured by Cochlear, Ltd. (Sydney, Australia), including the Nucleus CI24M, CI24R(CS), CI24R(CA), CI24RE (including Nucleus Freedom and CI422), and CI512 (Table 1).

### 2.3. Definition of Complications

In this study, major complications were defined as a permanent disability or an adverse event that required major surgical intervention or resulted in revision surgery [1], including permanent facial paralysis, meningitis, severe scalp flap infection, severe hematoma (needing revision), cholesteatoma, persistent CSF leakage, device failure, electrode misplacement, and magnet displacement. Minor complications were those that could be managed medically, including hematoma or seroma, soft-tissue infection, persistent otitis media, scalp folliculitis, transient dizziness or vertigo, change in taste, stitch infection, intermittent CSF gusher, and transient facial palsy.

### 2.4. Test Materials

Four open-set speech perception tests were used to examine the patients, including an easy sentence test, a difficult sentence test, a phonetically balanced (PB) word recognition test, and a Mandarin tone recognition test. The two sentence tests were designed based on the Central Institute for the Deaf (CID) Everyday Sentence test [18]. The easy sentence test included 15 sentences varying in length from two to ten words. Each sentence contained one to seven key words chosen from a corpus of words that were familiar to the subjects in their daily communication, such as “book” and “car” (File S1 in Supplementary Material available online at http://dx.doi.org/10.1155/2016/8962180). The difficult sentence test consisted of 20 sentences varying in length from two to twelve words. Each sentence embedded one to ten key words that were to be scored, but these key words were less familiar to children, such as “examine” and “dormitory” (File S2). The PB word recognition test, developed by Wang and Su [19], included 25 monosyllabic words (File S3). The Mandarin tone recognition test was developed and modified by Liu et al. [20]. The four Mandarin tones (flat, rising, dipping, and falling) were equally distributed throughout the word list.

Other than speech perception, the auditory receptive abilities and speech intelligibility of the patients were rated using the CAP and SIR scales, respectively. The CAP is an eight-point nonlinear and hierarchical rating scale. Its scores range from the lowest level (0) of being unaware of environmental sounds to the highest level (7) of having the ability to converse on the telephone with a familiar person (see Table S1). Its reliability has been demonstrated [21]. The SIR is a five-point nonlinear scale that reflects patient speech production intelligibility from the lowest level (1) of being unintelligible to the highest level (5) of being easily understood by all listeners (see Table S1). The reliability of the scale has been confirmed [22].

### 2.5. Test Procedures

The outcome of reimplantation was assessed using the pure tone audiometry (PTA), CAP, and SIR scales and four open-set speech perception tests with a CI only (not wearing a contralateral hearing aid). PTA or play audiometry tests were conducted in a sound-insulated booth (level of the background noise < 30 dB A) equipped with a two-channel clinical audiometer (GSI 61; Grason-Stadler Inc., Eden Prairie, MN). Mean hearing thresholds (0.5, 1, 2, and 4 kHz) with the CI were measured so as to show the auditory outcome with the CI. The CAP and SIR scales were rated by two speech therapists as a routine evaluation (Tables S1, S2). It was assessed every 3 months in the first year after mapping, every 6 months in the second year, and once annually until 5 years after implantation [15]. Speech perception tests were conducted in a sound-insulated booth. The stimulus level was controlled at 60 dB HL. The patients sat in front of the audiologist (at a distance of 1 m) and were asked to orally repeat the words or sentences they heard. The easy sentence and difficult sentence tests were scored based on how many key words the patient repeated correctly, as was the PB word test. The Mandarin tone recognition test was scored according to the tones only. A word would be counted correct as long as its tone was correctly repeated; the mistakes the patient made on the vowel or consonant were overlooked. The number of the correctly repeated items for each test was converted into percentages (% correct) for further analysis. To minimize learning effects, the testing lists were randomly selected. The answers were recorded for later evaluation, and the speech perception procedure was administered preoperatively and 3 months, 6 months, and 1 year postoperatively; not all children in this study underwent this test because of the age limit. However, in patients with reimplantation, some of the children already have the ability to take this testing. The following analysis was primarily based on the patients' scores before reimplantation, one year after implantation, and latest follow-up [23].

### 2.6. Statistical Analysis

Statistical analyses were performed using the SPSS software (ver. 17.0; SPSS, Inc., Chicago, IL, USA). Descriptive statistical analyses were conducted for cases with major complications and speech perception outcomes after reimplantation. Frequency measurements were carried out for the calculation of categorical data. The paired Wilcoxon signed-rank test and *t*-test were used to determine whether the scores obtained at different testing points differed significantly. A *χ* ^2^ test was used to examine the association between the occurrence of infection and the incision method (inverted J/minimally invasive). Significance was set at *P* < 0.05.

## 3. Results

From 1999 to 2014, a total of 589 patients received CIs by two surgeons in our medical center (Table 1). Among them, 511 (86.8%) were implanted before the age of 18 years and 78 (13.2%) after 18 years. Congenital hearing loss was found in 394 (66.9%) cases.

### 3.1. Major Complications

In total, 22 cases were found to have major complications, resulting in a major complication rate of 3.74% in our cohort (Table 2). Spontaneous device failure (*n* = 8; 1.36%) was one of the most common major complications and was mainly due to the malfunction of the Nucleus CI512 device (*n* = 7). When these seven cases were discarded, the rate of device failure decreased to 0.17% (*n* = 1) and that of major complications was lowered to 2.54% (*n* = 15). Excluding cases with spontaneous device failure, the major surgical complication rate was 2.38% (*n* = 14).

Infection, including severe scalp flap infection and mastoiditis, was the other common major complication (12 cases, 2.04%). Among the 255 patients who received a CI via an inverted J incision, 10 (3.92%) had infections after surgery. Among the 334 patients who underwent a minimally invasive incision, infection occurred only in 2 (0.60%). The *χ* ^2^ test showed that there was a significant association between the occurrence of infection and the incision method (*χ* ^2^ = 7.999, *P* = 0.005).

Although hematoma was usually considered a minor complication, one case in our cohort developed a severe hematoma that required revision surgery. Magnet displacement was found in only one case. None of the cases developed meningitis or permanent facial paralysis after CI surgery, and electrode misplacement did not occur in our cohort.

The significant association between the incision method and postoperative infection also explains the large difference between the infection rates in the Linkou and Kaohsiung/Xiamen branches (2.74% versus 0.53%): more surgeries were performed via a minimally invasive incision by the surgeon in Kaohsiung/Xiamen (82.45% of cases) than by the one in Linkou (44.64%). This shows that postoperative infection may be better prevented by a smaller incision line and hence a smaller wound.

### 3.2. Revision and Reimplantation Surgeries

In total, 18 (3.06%) patients underwent revision surgeries due to infection (*n* = 9), device failure (*n* = 8), or severe hematoma (*n* = 1; Table 3). The case with the severe hematoma received revision surgery to manage active bleeding from a subperiosteal pocket that persisted for 10 days after the primary operation but was not explanted or reimplanted. All eight cases with device failure (one with the Nucleus 24R(CS) and seven with the Nucleus CI512) also underwent reimplantation at our center. The operation was paid for by the national health system; the device was supplied by the CI manufacturer without charge. The other five cases with infection received reimplantation in our center, two on the ipsilateral side (R1 and R2) and three on the contralateral side (R3, R4, and R5).

In total, 13 patients underwent CI reimplantation at our medical center over the past 16 years (Table 4). All electrodes were inserted during reimplantation. Only one case was wearing a contralateral hearing aid after reimplantation. The mean time between initial implantation and reimplantation was 3.1 years (range, 0.8–12.1). Devices were replaced for the following reasons: hard device failure (8/13, 61.5%), soft device failure (0%), wound/flap infection (3/13, 23.1%), and device infection or extrusion (2/13, 15.4%). There was no reimplantation due to upgrade of the implant technology only.

### 3.3. Hearing and Speech Performance after Reimplantation

Patients reimplanted at another hospital or explanted but not reimplanted were excluded. The hearing and speech perception scores were collected from 13 patients to evaluate their reimplantation outcomes. The mean CAP and SIR scores obtained before and at least 1 year after reimplantation were 5.5 ± 1.1 versus 5.8 ± 0.7 and 3.7 ± 1.3 versus 4.5 ± 0.8 (mean ± SD), respectively (Table 4). The median SIR after reimplant use was 5, which was significantly better than preoperation (paired Wilcoxon signed-rank test, *P* = 0.009).

Four open-set speech perception scores were collected from 11 patients to evaluate their reimplantation outcomes (excluding cases R3 and R13, who did not take the tests). The results were collected 1 year after reimplantation. The mean scores of the Mandarin tone recognition test, easy sentence test, difficult sentence test, and PB word test obtained at 1 year after implantation were 62.7%, 81.3%, 70.0%, and 74.6%, respectively (Table 4). Only six patients received speech perception tests before reimplantation. The mean scores of the four tests obtained before and after reimplantation were 57.9% versus 58.3%, 80.3% versus 91.0%, 76.8% versus 76.7%, and 81.7% versus 83.3%, respectively (Table 4). There was no significant difference (*P* > 0.05) before and after reimplantation. Another 11 cases with age, sex, and operation age-matched CI recipients but without implantation were chosen as the control group (Table 5). Their latest speech perception scores were compared with those who underwent reimplantation. There was no significant difference between the experimental and control groups in the latest scores on the CAP, SIR, tone, easy sentence, difficult sentence, or word tests.

## 4. Discussion

Our review of 589 CI cases from 1999 to 2014 revealed 18 (3.06%) revision surgeries and 13 (2.2%) reimplantations, including 8 (1.4%) due to device failure. Wang et al. [2] report an overall revision/reimplantation rate of 7.6%/5.1% in 29 medical institutions. The relatively low rates may reflect the fact that only products manufactured by Cochlear Ltd. are used at our center. Most products used in the study are very stable except for the CI512. Among them, seven cases of a loss of hermetic seal occurred in the Nucleus CI512 implants; these were categorized as hard failures. Another reason is most likely because no soft device failure was noted in our center. We used the soft failure criteria established by the 2005 Consensus Development Conference for our CI population analysis [5]. No patient in our series experienced nonauditory complains such as facial nerve stimulation, ear pain, headache, tinnitus, or vertigo that was severe enough to undergo reimplantation surgery. Some cases with gradual or intermittent dips in performance improved after remapping and thus did fulfill the criteria of the 2005 Consensus. The soft failure rate in other series ranged from 15 to 56.4% of device failures [3, 10, 24, 25]. These data suggest that we should pay particular attention to Mandarin-speaking CI users requiring revision of their implants due to undesirable symptoms or decreasing performance of uncertain cause. Because the soft failure rate was very low at our center, in contrast to data reported from other countries, we are cautious and question whether there is some difference in our follow-up in this respect.

Recently, reported rates of revision and device failure in Mandarin-speaking CI users ranged from 1.7% to 5.9% and 0% to 2.4%, respectively [26–28]. In these studies (1822 cases), no Mandarin-speaking CI user underwent reimplantation due to soft device failure. After further analysis, we found that soft failure was suspected in four cases, including one case of facial stimulation, one case of poor auditory stimulation, and two cases with poor hearing result. Device explantation without reimplantation surgery was performed in these four cases, again not meeting the 2005 Consensus criteria. Soft device failure has rarely been reported in Mandarin-speaking CI users, calling the attention of surgeons and audiologists.

The reimplantation surgeries in our cohort were mainly due to device failure (of the Nucleus CI512) and infection. Full insertion of a new electrode array was accomplished in all of our patients; the auditory performance was improved or similar after reimplantation. Previous studies found that 79–86% of these patients regained speech perception ability shortly (≤1 year) after reimplantation and reported reattaining performance levels they reached before reimplantation [8, 17]. Speech perception scores used were closed- and open-set words or closed- and open-set sentences tests in previous studies. Both tests take a long time to administer, and patients may not regularly receive these speech tests for many years. This study is the first report of the use of CAP and SIR to evaluate outcomes before and after reimplantation. These real-life measures attract many clinicians and audiologists because they are easily administered in a clinical setting, such as a cochlear implant center, and can be readily applied to young deaf children each year, regardless of their intellectual or other characteristics [29]. Our analyses indicated that the CI patients had restored CAP scores within the first year after reimplantation, whereas the SIR scores were significantly better than preoperation. The mean time to reimplantation was 3.1 years in our series. A previous study demonstrated that the patients had steadily improved CAP scores in the first 3 years after initial implantation and reached a plateau after 3 years of implant use, whereas the SIR had the potential to show improvement after 3 years of implant use [15]. Indeed, progress may continue even after 10 years of use [30].

SIR is related to articulation problems and thus could be improved with continuous practice in speaking. With more practice, the SIR score is expected to become higher, although the scores from the CAP or objective word test do not improve much after some years of implant use. Thus, it is reasonable to expect that the SIR of the patients in the present study will continue to improve after reimplantation.

Mandarin is a tonal language, in which tone exploits a change in the fundamental frequency (F0) pattern within the same phonemic segment distinguishing word meaning. In Mandarin Chinese, the patterns vary in 1 of 4 ways, thus 4 tones: (1) flat and high, (2) rising, (3) falling and then rising, and (4) falling [31]. However, this information is usually largely discarded in most current CI systems. There are only 4 patterns of tone in Mandarin, which is far fewer than the number of speech sounds. Consistent with previous study [23], tone identification score was also poorer than word and sentence perception in our series. We found that most patients regained tone recognition ability shortly (about 1 year) and the performances were either improved or similar after reimplantation.

For patients with wound infection following surgery, we suggest that surgeons remove the implant immediately once they find that explantation is inevitable. This allows reimplantation in the ipsilateral ear when the patient recovers from the infection. By ipsilateral reimplantation, the auditory benefit brought by the primary implantation can be preserved, and the surgical procedure will be less complicated. The patient can thus still possibly receive a second implant on the contralateral side in the future. If the surgeon has to reimplant in the contralateral ear due to anatomical variations in the middle ear or mastoid process (as a consequence of delayed treatment of infection), the patient may need more time to reattain the performance level reached before reimplantation because the contralateral ear has not received any stimulation for a long time. Some surgeons have proposed device relocation as an alternative in treating infection [32]. However, the outcome may not be satisfactory due to biofilms [33]. Infection can develop at the new site and, eventually, removal of the device may be necessary.

As discussed above, many complications can be effectively prevented by experience. It is thus of great importance that cochlear implant centers and manufacturers follow patients' postimplant outcomes regularly to keep a reliable record and learn from experience. Furthermore, considering that CI is now performed on very young children, experienced audiologists at the cochlear implant center need to pay close attention to their performance and function after surgery because these children are still incapable of fully expressing themselves, which may result in delayed treatment.

Although the positive effect of education on cognitive skills is currently widely accepted among adults and elderly people, the impact of education on auditory processing has rarely been evaluated [34, 35]. Until recently, the variable “educational level” was not routinely taken into consideration at the time of auditory processing diagnoses. Murphy et al. (2016) suggested that a higher educational level also correlated with improved auditory processing skills, including speech-in-noise test performance [36]. In our series, the hearing and speech perception scores were collected from 10 children and 3 adults (R1, R8, and R10) to evaluate their reimplantation outcomes. In the future, we should take education level into consideration, especially in adults and elderly people.

Due to the retrospective design, minor differences in the surgical methods and postoperative assessments adopted by the two surgeons in this study may not have been taken into consideration. It is also difficult to compare the complication and revision rates between studies as the numbers of patients, types of devices, and follow-up periods were all different. This calls for an international consensus on reporting complications to allow better comparability. Finally, follow-up was performed after various intervals, and it is likely that the follow-up protocols differed greatly. For example, some patients did not undergo open speech perception tests. Despite the limitations, however, CI is widely considered a safe procedure, as manifested in our low rates of revision and device failure, and the outcomes of reimplantation were satisfactory in most patients.

## 5. Conclusions

Cochlear implantation is a safe procedure, with low rates of postsurgical revision and device failure. Mandarin-speaking patients who received reimplantation are capable of restoring auditory performance and speech intelligibility after surgery. Regular follow-up of postimplant outcomes is of critical importance to allow prompt treatment of infections and prevent the need for reimplantation. Device soft failure was rare in our series, calling special attention to Mandarin speaking CI users requiring revision of their implants due to undesirable symptoms or decreasing performance of uncertain cause.

## Supplementary Material

S1 File. Easy sentence list for the speech perception test (English translation).
S2 File. Difficult sentence list for the speech perception test (English translation).
S3 File. Phonetically-balanced monosyllabic word list for the speech perception test.
S1 Table. Criteria of the Categorical Auditory Performance (CAP) and Speech Intelligibility Rating (SIR) scales.

## Figures and Tables

**Table 1:** Demographic backgrounds of the cases.

	Linkou	Kaohsiung & Xiamen	Total
*Total number of CI patients*	401	188	589
*Gender (male/female)*	210/191	102/86	312/277
*Implanted ear (right/left)*	186/215	103/85	289/300
*Age at implantation (years)*			
≤3.0	160	80	240
3.1–5.0	88	49	137
5.1–18.0	100	34	134
>18.0	53	25	78
*CI device*			
CI24M	61	7	68
CI24R (CS, CA)	92	81	173
CI24RE (Freedom)	194	75	269
CI512	43	14	57
CI422	11	11	22
*Incision method*			
Inverted J	222	33	255
Minimally invasive	179	155	334
*Etiology*			
Congenital	243	144	387
LVAS	61	13	74
Progressive	21	6	27
Inner ear anomaly	28	5	33
Waardenburg syndrome	8	3	11
Meningitis	7	1	8
Sudden deafness	3	4	7
Multiply handicapped	5	3	8
Other	25	9	34

**Table 2:** Causes of major complications following cochlear implantation.

	Linkou	Kaohsiung & Xiamen	Total
*Major complications*			
Device failure	7	1	8
Hematoma needing revision	0	1	1
Permanent facial paralysis	0	0	0
Mastoiditis	2	1	3
Severe scalp flap infections	9	0	9
Meningitis	0	0	0
Retraction pocket with cholesteatoma	0	0	0
Persistent CSF leakage	0	0	0
Electrode misplacement/pull-out	0	0	0
Magnet displacement	1	0	1
Total number of cases	19	3	22
Rate of major complications	4.74%	1.60%	3.74%

**Table 3:** Causes of revision surgery following cochlear implantation.

	Linkou	Kaohsiung & Xiamen	Total
*Device failure*	7	1	8
Hard failure	7	1	8
Soft failure	0	0	0
*Medical complications*	9	1	10
Infection	9	0	9
Hematoma	0	1	1
*Device update*	0	0	0
Total number of cases	16	2	18
Rate of CI revision	3.99%	1.06%	3.06%

**Table 4:** Patients undergoing reimplantation and hearing/speech performance before and after surgery.

Number	Cause	Implant/SP	Age at CI (years)	Hearing and speech before reimplantation							Hearing and speech after reimplantation							
				PTA (dB HL)	CAP	SIR	Tone recognition (%)	Easy sentence (%)	Difficult sentence (%)	PB word (%)	Interval (years)	PTA (dB HL)	CAP	SIR	Tone recognition (%)	Easy sentence (%)	Difficult sentence (%)	PB word (%)
R1	Infection (flap)	CI24RE/CP(810)	6.5	32.5	7	5	n/a	n/a	n/a	n/a	12.7	25	6	5	80	92	95	36
R2	Infection (device)	CI24M/Sprint	1.5	27.5	6	5	n/a	n/a	n/a	n/a	4.3	30.0	6	5	88	84	69	88
R3	Infection (flap)	CI24R(CS)/Esprit	5.0	33.8	4	2	n/a	n/a	n/a	n/a	1.9	31.3	4	3	n/a	n/a	n/a	n/a
R4	Infection (device)	CI24R(CS)/CP(810)	4.3	32.5	7	5	85	100	95	84	4.1	28.8	7	5	74	100	95	84
R5	Device failure	CI24R(CS)/CP(810)	3.1	33.8	4	2	n/a	n/a	n/a	n/a	1.4	23.8	6	4	53	32	24	36
R6	Device failure	CI24RE/CP(810)	2.7	30.0	6	4	53	100	92	98	3.9	31.3	6	5	76	98	91	84
R7	Device failure	CI24RE/CP(810)	3.1	28.8	4	2	n/a	n/a	n/a	n/a	1.3	25	5	3	41	40	28	68
R8	Device failure	CI24RE/CP(810)	51.5	21.3	7	5	35	98	94	60	0.8	22.5	6	5	58	100	98	88
R9	Device failure	CI24RE/CP(810)	2.8	26.3	6	3	45	26	28	76	2.5	26.3	6	5	43	82	24	92
R10	Device failure	CI24RE/CP(810)	26.3	31.3	6	5	84	98	98	100	1.8	32.5	6	5	85	100	93	80
R11	Device failure	CI24RE/CP(810)	2.6	25.0	5	3	46	60	54	72	2.5	23.8	6	4	15	66	59	72
R12	Infection (flap)	CI24RE/CP(810)	1.8	26.3	5	3	n/a	n/a	n/a	n/a	1.6	27.5	6	5	79	100	94	92
R13	Device failure	CI24RE/CP(810)	1.6	29.0	5	4	n/a	n/a	n/a	n/a	1.2	29.0	6	5	n/a	n/a	n/a	n/a

SP, speech processor; CI, cochlear implantation; PTA, mean pure tone audiometry thresholds (0.5, 1, 2, and 4 kHz); CAP, categorical auditory performance; SIR, speech intelligibility rating; PB word, phonetically balanced word perception; n/a, not available.

**Table 5:** Patients undergoing cochlear implantation and hearing/speech performance after surgery.

Number	Implant/SP	Age at CI (years)	Hearing and speech after CI implantation					
			CAP	SIR	Tone recognition (%)	Easy sentence (%)	Difficult sentence (%)	PB word (%)
C1	CI24M/CP(810)	5.7	7	5	75	100	94	80
C2	CI24M/CP(910)	1.8	6	5	55	98	87	80
C3	CI24R(CS)/Sprint	4.2	6	5	80	48	39	12
C4	CI24R(CS)/CP(910)	3.2	6	5	30	40	16	44
C5	N512/CP(810)	2.0	6	5	94	100	90	100
C6	N512/CP(810)	3.1	6	5	96	100	90	100
C7	N512/CP(810)	48.2	6	5	68	100	97	40
C8	N512/CP(810)	2.7	6	5	61	100	67	100
C9	CI24RE/Freedom	25.3	6	5	83	100	97	64
C10	N512/CP(810)	2.6	5	4	20	91	67	80
C11	CI24RE/CP(810)	2.4	6	5	89	100	95	88
