# Supplementary material for: Comparisons of Auditory Performance and Speech Intelligibility after Cochlear Implant Reimplantation in Mandarin-Speaking Users
Source: Biomed Res Int. 2016 Jun 16;2016:8962180. doi: 10.1155/2016/8962180 (PMC4927948; doi:10.1155/2016/8962180)
Supplement: Supplementary file 1 — S1 File. Easy sentence list for the speech perception test (English translation). S2 File. Difficult sentence list for the speech perception test (English translation). S3 File. Phonetically-balanced monosyllabic word list for the speech perception test. S1 Table. Criteria of the Categorical Auditory Performance (CAP) and Speech Intelligibility Rating (SIR) scales. [file 8962180.f1.zip › 8962180 - S1 Table.docx]

S1 Table. Criteria of the Categorical Auditory Performance (CAP) and Speech Intelligibility Rating (SIR) scales.

| Rating scale | CAP Criteria | SIR Criteria |
| --- | --- | --- |
| 7 | Use of telephone with known listener |  |
| 6 | Understanding of conversation without lip-reading |  |
| 5 | Understanding of common phrases without lip-reading | Connected speech is intelligible to all listeners. Child is understood easily in everyday contexts |
| 4 | Discrimination of some speech sounds without lip-reading | Connected speech is intelligible to listener who has a little experience of deaf person’s speech |
| 3 | Identification of environmental sounds | Connected speech is intelligible to a listener who concentrates and lip-reads |
| 2 | Response to speech sounds | Connected speech is unintelligible. Intelligible speech is developing in single words when context and lip-reading cues are available |
| 1 | Awareness of environmental sounds | Connected speech is unintelligible. Pre-recognizable words in spoken language, primary mode of communication may be manual |
| 0 | No awareness of environmental sounds |  |
