# Supplementary material for: Comparisons of Auditory Performance and Speech Intelligibility after Cochlear Implant Reimplantation in Mandarin-Speaking Users
Source: Biomed Res Int. 2016 Jun 16;2016:8962180. doi: 10.1155/2016/8962180 (PMC4927948; doi:10.1155/2016/8962180)
Supplement: Supplementary file 1 — S1 File. Easy sentence list for the speech perception test (English translation). S2 File. Difficult sentence list for the speech perception test (English translation). S3 File. Phonetically-balanced monosyllabic word list for the speech perception test. S1 Table. Criteria of the Categorical Auditory Performance (CAP) and Speech Intelligibility Rating (SIR) scales. [file 8962180.f1.zip › 8962180 S1-3 files.doc]

**S1 File.** Easy sentence list for the speech perception test (English translation). The key words are underlined.
(1) This book is good.
(2) Have you brought all your stuff?
(3) Be careful when driving the car.
(4) It does not matter.
(5) Turn off the television.
(6) Where is the newspaper?
(7) Excuse me, is the boss there?
(8) That coat looks really good.
(9) I am having a headache.
(10) Tomorrow is a holiday. Do you want to go out?
(11) It seems that I am having a fever.
(12) Please don’t smoke.
(13) The weather is quite nice today.
(14) How many brothers and sisters do you have?
(15) How many people are there in your family?

**S2 File.** Difficult sentence list for the speech perception test (English translation). The key words are underlined.
(1) Can you hear what he is saying?
(2) How is your health condition?
(3) How about it?
(4) We will go there right after having dinner.
(5) Who is in there?
(6) Very busy.
(7) This movie is very good.
(8) We often go to the park.
(9) This building is taller than that one.
(10) The doctor comes to carry out examinations on him every day.
(11) Many books are put on the table.
(12) I am so tired.
(13) Is sister busily working?
(14) I am a student.
(15) That novel is very interesting.
(16) There will be a movie today evening.
(17) He has graduated for about two years.
(18) When he is anxious, he cannot even speak a word.
(19) Sit down, please!
(20) It is summer now.

**S3 File.** Phonetically-balanced monosyllabic word list for the speech perception test. Pinyin characters are used. English translations are parenthesized.

| bà (dad) | zhǐ (only) | nǔ (effort) | ling (bell) | qì (air) |
| --- | --- | --- | --- | --- |
| nioú (cow) | zuǒ (left) | jìn (nearby) | yén (salt) | gòu (enough) |
| jiěn (minus) | fù (father) | kāi (open) | zhú (bamboo) | rù (enter) |
| jiǎng (talk) | cīng (star) | lí (pear) | zhè (this) | zhàn (battle) |
| yā (duck) | yào (want) | yú (fish) | jiěh (sister) | guó (nation) |
